# Glycosylation of ceramide synthase 6 is required for its activity

**DOI:** 10.1016/j.jlr.2024.100715

**Published:** 2024-11-26

**Authors:** Alexandra J. Straus, Grace Mavodza, Can E. Senkal

**Affiliations:** 1 Department of Biochemistry and Molecular Biology, Virginia Commonwealth University School of Medicine, Richmond, VA, USA; 2 C. Kenneth and Dianne Wright Center for Clinical and Translational Research, Virginia Commonwealth University School of Medicine, Richmond, VA, USA; 3 Massey Comprehensive Cancer Center, Virginia Commonwealth University School of Medicine, Richmond, VA, USA

**Keywords:** ceramide, sphingolipids, ceramide synthase, CerS6, lipidomics

## Abstract

Sphingolipids play key roles in membrane structure and cellular signaling. Ceramide synthase (CerS)-generated ceramide is implicated in cellular stress responses and induction of apoptosis. Ceramide and other sphingolipids are linked to the induction of ER stress response pathways. However, the mechanisms by which ceramide modulates ER stress signaling are not well understood. Here, we show that the ER stress inducer brefeldin A (BFA) causes increased glycosylation of CerS6, and that treatment with BFA causes increased endogenous ceramide accumulation. To our surprise, we found that CerS6 activity was not affected by BFA-induced glycosylation. Instead, our data show that basal glycosylation of CerS6 at Asn18 is required for CerS6 activity. We used a robust HCT116 CRISPR-Cas9 CerS6 KO with reintroduction of either WT CerS6 or a mutant CerS6 with a point mutation at asparagine-18 to an alanine (N18A) which abrogated glycosylation at that residue. Our data show that cells stably expressing the N18A mutant CerS6 had significantly lower activity in vitro and in situ as compared to WT CerS6 expressing cells. Further, the defective CerS6 with N18A mutation also had defects in GSK3β, AKT, JNK, and STAT3 signaling. Despite being required for CerS6 activity, Asn18 glycosylation did not influence ER stress response pathways. Overall, our study provides vital insight into the regulation of CerS6 activity by posttranslational modification at Asn18 and identifies glycosylation of CerS6 to be important for ceramide generation and regulation of downstream cellular signaling pathways.

Sphingolipids, a family of lipids containing long chain sphingoid bases, have been implicated in a multitude of cellular signaling pathways and are crucial for membrane structure and fluidity (1, 2, 3, 4). Ceramide, the central sphingolipid molecule, can be generated de novo at the ER beginning with the condensation of palmitoyl-CoA and serine by serine palmitoyl transferase (SPT) to form 3-ketodihydrosphingosine (3KDS) (5, 6). 3KDS is subsequently converted to dihydrosphingosine (dhSph) by 3-ketodihydrosphingosine reductase (3KDSR). DhSph is then acylated by one of six ceramide synthase (CerS) isoforms to generate dihydroceramide. The six CerS isoforms, CerS1-6, exhibit preferences for acyl-CoA of different chain lengths to produce dihydroceramides of specific chain lengths. For example, CerS5 and CerS6 preferentially generate C_14:0_ and C_16:0_-ceramides, while CerS1 primarily generates C_18:0_-ceramide (7, 8, 9). Dihydroceramide is subsequently desaturated to produce ceramide, which can then be transported to the Golgi for generation of sphingomyelin or glucosylceramide by ceramide transport protein or vesicular transport, respectively (10). In addition to the de novo sphingolipid pathway, ceramide can be generated through sphingomyelin (SM) hydrolysis by sphingomyelinase or through the salvage pathway in which complex sphingolipids (SM and glycosphingolipids) are transported and degraded in the lysosome to generate sphingosine which is then transported to the ER where it can be used by CerS enzymes to produce ceramide (11).

Ceramides have roles in mediating various fundamental cellular processes such as apoptosis (12), regulation of cell cycle (13), inflammatory responses (14), and other cellular stress responses (15), including ER stress response (16). ER stress response occurs primarily due to an excess of unfolded or misfolded proteins in the ER, causing activation of the unfolded protein response (UPR) which will either activate expression of genes required to restore ER homeostasis or, if necessary, apoptosis (17). UPR involves three signaling pathways: inositol-requiring protein-1 (IRE1); activating transcription factor-6 (ATF6), and protein kinase RNA (PKR)-like ER kinase (PERK). In their inactive state, IRE1, PERK, and ATF6 are bound to the ER chaperone binding immunoglobulin protein (BiP/GRP78). However, upon accumulation of unfolded proteins, BiP detaches from the stress sensors and binds to the unfolded proteins, allowing each of the stress sensors to induce signaling cascades to either restore ER homeostasis or induce apoptosis. Upon dissociation from BiP, IRE1 can splice the transcription factor X-box binding protein 1 (XBP1), and also induce regulated IRE1-dependent decay (RIDD) to restore ER homeostasis. Prolonged ER stress can induce apoptosis via c-Jun N-terminal kinase (JNK) through its activation by IRE1 (18). PERK, on the other hand, phosphorylates eukaryotic initiation factor 2a (eIF2a), which then inhibits protein translation, and ATF4, which activates transcription of the proapoptotic C/EBP homologous protein (CHOP) (19). Lastly, upon dissociation from BiP, ATF6 is transported to the Golgi apparatus where it is cleaved and translocated to the nucleus where it regulates UPR target genes as a transcription factor (20).

While accumulation of unfolded proteins is the primary cause of UPR, recent studies have shown that disruption of Ca^2+^ homeostasis and alterations to lipid, including sphingolipid, composition and microdomains on the ER can induce UPR as well (16, 21, 22, 23). Because sphingolipids are known to alter membrane properties and signaling functions, sphingolipids within the ER can regulate ER stress sensor activation (24). However, the mechanisms by which sphingolipids regulate ER stress response, and vice versa, are not well understood.

In this work, we investigated the effect of three known ER stress inducers—brefeldin A (BFA), thapsigargin, and tunicamycin—on the sphingolipid metabolic pathway. We found that BFA, but not thapsigargin or tunicamycin, induces accumulation of C_16:0_-ceramide. In addition, BFA treatment leads to endo-H resistant glycosylation of CerS6. While BFA caused additional glycosylation of CerS6, this did not directly affect its activity. Significantly, we found that basal glycosylation of CerS6 at Asn18 is required for its activity. Importantly, we also show that the change in basal CerS6 activity due to loss of glycosylation can cause changes in cellular signaling events such as the phosphorylation of several proteins important for signaling events in cells. Taken together, we show that CerS6 glycosylation is an important posttranslational modification that regulates CerS6 activity, which may be leveraged for therapeutic use in pathologies where CerS6 activity is elevated.

## Materials and Methods

### Cell lines and growth conditions

HCT116 colorectal carcinoma cells were originally provided by Dr Richard J. Youle (NINDS, NIH, USA). Cells were grown in DMEM (cat# 10-017-CV, Corning) supplemented with 10% FBS (Cat# S12495, lot:K20158, Bio-Techne) without antibiotics at 37°C humidified incubator with 5% CO_2_. Stable expression of plasmids in HCT116 cells was performed as described previously (25). Possible mycoplasma contaminations were monitored regularly by MycoAlert mycoplasma detection kit (Lonza, cat#LT07-318).

### Generation of HCT116 CRISPR-Cas9 KO cells

CRISPR-Cas9 mediated KO of CerS6 in HCT116 cells was performed using CRISPRevolution sgRNA EZ Kit and Cas9 2NLS Nuclease (Synthego). The sequence of guide RNA used was ggcucccgcacaaugucacc. Cas9 and sgRNA were delivered into the cells using Lipofectamine CRISPRMAX (Thermo Fisher Scientific, cat#CMAX00001). After 48 h, cells were diluted into a 96-well plate and after sufficient cell growth, individual colonies were screened for the loss of CerS6 using immunoblotting.

### Plasmid constructs

V5- and FLAG-tagged WT-CerS6 in pLenti6.3 vector were used as described (25). The N18A mutant CerS6 was generated using the Q5 Site Directed Mutagenesis kit (cat# E0554S, New England BioLabs) as described by the manufacturer using the forward primer 5′GCTCCCGCACGCTGTCACCTGG3′ and the reverse primer 5′CAAAACCTCTCGTTCCAG3’.

### Determination of cell growth

Cellular growth after treatments was determined by 3-(4,5-dimethyl-2-767 thizolyl)-2,5-diphenyl-2H-tetrazolium bromide assay, as described previously (26). For cell growth assays, cells were treated with BFA (Cayman Chemicals, cat#20350-15-6) at 50 ng/ml (178 nM), thapsigargin (Cayman Chemicals, cat#67526-95-8) at 50 nM, or tunicamycin (Cayman Chemicals, cat#11445) at 0.5 μg/ml (768 nM). Cells were treated for 24 h for cell growth assays or 16 h for all other experiments.

### Western blotting

Total cell lysates were mixed with 2X sample buffer (200 mM Tris-HCl pH 6.8, 40% glycerol, 8% SDS, 400 mM DTT, and 0.05% bromophenol blue). The samples were boiled for 5 min and resolved in 4%–20% Tris-HCl polyacrylamide gels (Thermo Fisher Scientific, Waltham, MA). After transferring the proteins to PVDF membranes (Bio-Rad, Hercules, CA), the blots were blocked with 5% nonfat milk in 1X Tris buffered saline with 0.3% Tween-20. The blots were incubated with the primary antibodies overnight at 4°C on a platform rocker. After washes with 1X Tris buffered saline with 0.3% Tween-20 and incubation with HRP-labeled secondary antibodies, the blots were washed for a final time and developed using enhanced chemiluminescence (Thermo Fisher Scientific). The blots were imaged either via X-ray films or using the Azure 600 Imager. The primary antibodies used in the study were as follows: anti-phospho-IRE1 (Novus, cat#NB100-2323); anti-total IRE1a (Proteintech, cat#27528-1-AP); anti-phospho-PERK(S719) (Proteintech, cat#29546-1-AP); anti-total PERK (Proteintech, cat#20582-1-AP); anti-XBP1 (Proteintech, cat#24868-1-AP); anti-Chop (Proteintech, cat#24169-1-AP); anti-actin (Proteintech, cat#66009-1-Ig); anti-CerS6 (Novus, cat#H00253782-M01); anti-GRP78 (Sigma-Aldrich, cat#G9043-200ul). The secondary antibodies used were goat anti-mouse (Jackson ImmunoResearch, cat#115-035-003) and goat anti-rabbit (Jackson ImmunoResearch, cat#111-035-003. All primary antibodies were used at 1:1000 dilutions, and secondary antibodies were used 1:5000 dilutions.

### LC/MS-MS analysis

Targeted sphingolipidomics was done by LC/MS analysis as described previously (27). Lipid measurements were normalized to total protein amounts for each sample.

### Immunofluorescence microscopy

HCT116 cells were plated in 35 mm confocal dishes (cat# P35GC-1.5-10-C, MatTek Corp.) with center wells. The following day the cells were treated with BFA. Sixteen hours later, immunofluorescence confocal imaging was carried out as described (25) using anti-calreticulin (Sigma-Aldrich, cat#C4606), anti-GM130 (Cell Signaling, cat#12480), or anti-CerS6 (Novus, cat#VAP97XC-M01) antibodies. Nuclei were stained using 4',6-diamidino-2-phenylindole (Abcam, cat#ab104139).

### In vitro dephosphorylation assays

Dephosphorylation of proteins in HCT116 cell lysates was performed using lambda phosphatase (New England BioLabs, cat#P0753S) according to the manufacturer’s instructions. Briefly, 4 μg of total protein in 3 μl 10X New England Biolabs buffer for protein metallo phosphatases, 3 μl 10 mM MnCl_2_, and diH_2_O were combined to make a total reaction volume of 30 μl. Next, 1 μl of lambda protein phosphatase was added to the reaction mixture, and the mixture was incubated at 30°C for 30 min. Controls for each reaction omitted the lambda protein phosphatase which was replaced by 1 μl diH_2_O. Ten microliters of the mixture was then resolved on a 7.5% SDS-PAGE gel (Bio-Rad, cat#5671024) and Western blotting was carried out.

### In vitro deglycosylation assays

Deglycosylation of proteins in HCT116 cell lysates was achieved using either PNGase F (New England BioLabs, cat#P0704S) or Endo H (New England BioLabs, cat#P0702S) recombinant proteins according to the manufacturer’s instructions. Briefly, for PNGase F treatment in denaturing conditions, 4 μg of protein was added to 1 μl of 10X glycoprotein denaturing buffer and diH_2_O to make 10 μl reaction volume. The mixture was then heated at 100°C for 10 min, and then chilled on ice for 10 s. Next, 2 μl 10X glycobuffer, 2 μl 10% NP-40, 6 μl diH_2_O, and 1 μl PNGase F were added, and the mixture was gently mixed. The mixture was then heated at 37°C for 1 h, and the samples were resolved on a 7.5% SDS-PAGE gel. For Endo H treatment, 4 μg protein lysate was combined with 1 μl 10X glycoprotein denaturing buffer and diH_2_O to make a volume of 10 μl reaction mixture. The mixture was then heated at 100°C for 10 min Briefly, 2 μl 10X glycobuffer 3, 1 μl Endo H, and diH_2_O were added to make 20 μl total. The mixture was then heated at 37°C for 1 h, and the sample was resolved on a 7.5% SDS-PAGE gel.

### In vitro and in situ CerS activity assay

In vitro CerS activity assays were performed as described previously (28) with minor modifications. Briefly, cells were mechanically homogenized in 25 mM Tris-HCl pH 7.4, 25 mM KCl, 2 mM MgCl2, and 250 mM sucrose supplemented with protease inhibitor cocktail (K1007, APExBIO). Total microsomes were isolated using centrifugation at 100,000 *g* for 1 h at 4°C. For activity assays after deglycosylation, microsomes were treated with PNGase F according to the vendor’s protocol for nondenaturing conditions and heated to 37°C for 30 min. Microsomes were resuspended in homogenization buffer and incubated with 15 μM NBD-Sphingosine (cat# 25348, Cayman Chemical), 20 μM defatted BSA, and 50 μM fatty acyl-CoA (cat# P9716 (palmitoyl-CoA)) in a 100 μl reaction at 37°C. Reactions were terminated by addition of chloroform/methanol (2:1 v/v). The phases were separated by centrifugation at 3000 *g* for 10 min at 4°C. The bottom phase was transferred into a new glass vial, and the lipids were dried under N_2_ stream. Lipids were resuspended in methanol and transferred into HPLC vials for injection. The fluorescent NBD-Sph and NBD-ceramide from the reactions were detected and measured using a Thermo Fisher Scientific Vanquish HPLC coupled with a fluorescence detector. In situ CerS activity was measured by 17C-Sphingosine labeling as described (29). Briefly, cells were incubated with 1 μM 17C-Sphingosine for 15 min and its incorporation into ceramide in the cells was determined by LC/MS.

### Analysis of XBP1 mRNA splicing

Loss of *Pst*I restriction site upon splicing of XBP1 mRNA was determined using RT-PCR as described (30). After isolation of total RNA and synthesis of complementary DNA, XBP1 mRNA was amplified using the forward primer 5′-GGAGTTAAGACAGCGCTTGG-3′ and the reverse primer 5′-TGAGAGGTGCTTCCTCGATT-3′. The resulting PCR product was purified, digested with *Pst*I, and separated on a 1.5% agarose gel. DNA was then visualized by SYBR-Safe staining under ultraviolet light using the Gel-Doc system (Bio-Rad).

### Proteome profiler phospho-kinase analysis

Changes to protein kinase phosphorylation were analyzed using the Proteome Profiler Human Phospho-Kinase Array Kit (ARY003C, R&D Systems) according to kit instructions. Protein concentrations of cell lysate were quantitated by Bradford analysis, and images were quantitated using ImageJ (NIH; https://imageJ.net/ij/).

### Statistical analyses

All data are presented as means ± SD of at least three independent studies (n ≥ 3). Group comparisons were performed with two-tailed unpaired t-tests for comparison of two groups, and with two-way ANOVA for comparison of more than two groups with Tukey’s multiple comparison tests using GraphPad Prism software, version 9.4.1. (https://www.graphpad.com), *P* < 0.05 was considered significant.

## Results

### Thapsigargin, tunicamycin, and brefeldin A induce ER-stress and cell death

**Fig. 1:**
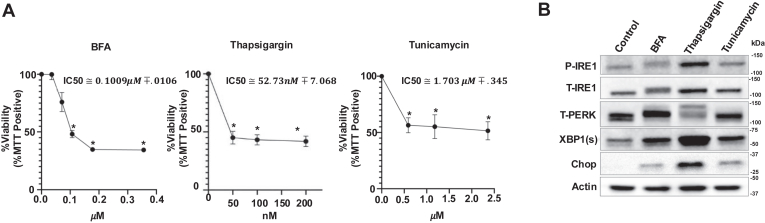
BFA, thapsigargin, and tunicamycin induce ER stress. A: HCT116 cells were treated with increasing concentrations of brefeldin A (BFA), thapsigargin, or tunicamycin for 24 h and cell viability was measured via MTT analysis. n = 3 (Three independent experiments with technical duplicates). Data represent mean ± SD. Statistical analysis was done by two-tailed unpaired *t* test, ∗*P* < 0.05. IC50 values calculated using Prism. B: Activation of UPR was determined by Western blotting after treatment with BFA (50 ng/ml (178 nM)), thapsigargin (50 nM), or tunicamycin (0.5 μg/ml (768 nM)) for 16 h. Representative blots from three experiments are presented. UPR, unfolded protein response; MTT, 3-(4,5-dimethyl-2-767 thizolyl)-2,5-diphenyl-2H-tetrazolium bromide; BFA, brefeldin A.

To study the effects of ER stress on sphingolipid metabolism, we used three ER-stress inducers: BFA, thapsigargin, and tunicamycin, all of which work through different molecular mechanisms. Thapsigargin induces ER stress by inhibiting the sarco/ER Ca^2+^-ATPase (SERCA) (31). Tunicamycin induces the UPR by inhibiting N-linked glycosylation in proteins (32, 33), and BFA disrupts the Golgi structure, triggering ER stress pathways (34). We first treated HCT116 colon cancer cells with increasing concentrations of the ER-stress inducers. Each compound induced cell death at the IC_50_ of about 0.1009 μM, 52.73 nM, and 1.703 μM for BFA, thapsigargin, and tunicamycin, respectively (Fig. 1A). Further, all three compounds resulted in activation of UPR pathways at the concentrations they were used. They all induced accumulation of CHOP, spliced XBP1, phosphorylation of IRE1α, and PERK, indicated by band shift, suggesting the induction of ER stress following the treatments (Fig. 1B).

### BFA induces accumulation of C_16:0_-ceramide

**Fig. 2:**
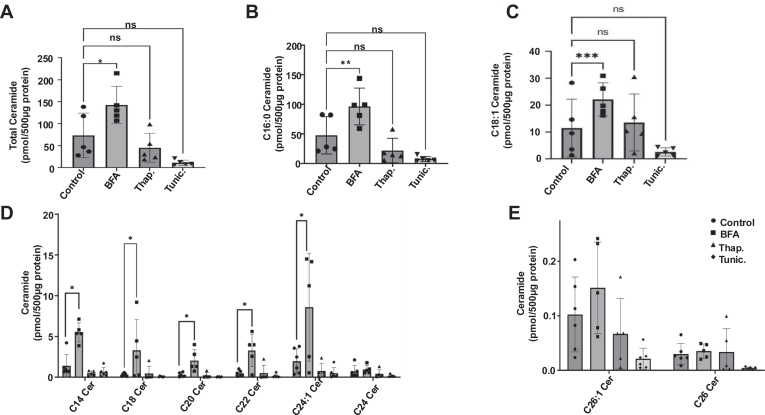
BFA treatment induces ceramide accumulation. A–E: Sphingolipid levels were measured in HCT116 cells by LC/MS as described in Materials and Methods. Cells were treated with BFA (50 ng/ml (178 nM)), thapsigargin (50 nM), or tunicamycin (0.5 μg/ml (768 nM)) for 16 h. Lipid abundance was normalized to 500 μg protein as measured by a Bradford assay. n = 3 (Three independent experiments with technical duplicates) Data represent mean ± SD. Statistical analysis was done by a two-way ANOVA with Tukey’s multiple comparison test ∗*P* < 0.05, ∗∗*P* < 0.005, ∗∗∗*P* < 0.001. BFA, brefeldin A.

To investigate the changes in the sphingolipid metabolism following ER-stress induction, we next analyzed the sphingolipid levels upon BFA, thapsigargin, and tunicamycin treatments using LC/MS. While thapsigargin and tunicamycin did not cause any significant changes in the ceramide abundance (Fig. 2A–E), BFA induced significant accumulation of both C_16:0_-ceramide and C_18:1_-ceramide as well as total ceramides (Fig. 2A–C). Specifically, C_16:0_-ceramide levels, the most abundant ceramide species in HCT116 cells, significantly increased following BFA treatment (Fig. 2B). We also observed significantly elevated C_14:0_, C_18:0_, C_20:0_, C_22:0_, and C_24:1_ ceramide levels after BFA treatment (Fig. 2D). There were not any significant changes in monohexosylceramide species in any of the three treatments (Supplemental Fig. S1). Of note, the monohexosylceramide measurements include both glucosylceramide species, synthesized in the Golgi, as well as galactosylceramide species, synthesized in the ER (35). Given that BFA is known to cause the collapse of Golgi into the ER, it is possible that there are changes in glucosyl- or galactosylceramide species that are not indicated by the bulk monohexosylceramide measurements shown here. There were slight, but significant decreases in C_16:0,_ C_20:0_, and C_22:0_-sphingomyelin levels following treatment with BFA (Supplemental Fig. S2). Further, there were no significant differences in the total levels of monohexosylceramide or total sphingomyelin after treatment with any of the ER-stress inducers (Supplemental Figs. S1B and S2B). Together, these results suggest that C_16:0_-ceramide may play a role in ER-stress following BFA treatment.

### BFA induces additional glycosylation of CerS6

**Fig. 3:**
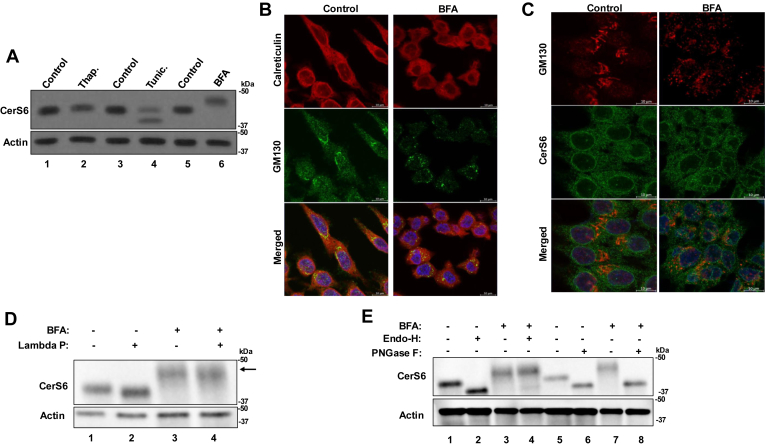
BFA treatment induces Endo-H resistant additional glycosylation of CerS6. A: CerS6 protein was detected by Western blotting after treatment with BFA (50 ng/ml (178 nM)), thapsigargin (50 nM), or tunicamycin (0.5 μg/ml (768 nM)) for 16 h. Representative blots from three experiments are presented. B: ER and Golgi were visualized by immunofluorescence confocal laser scanning microscopy by costaining of cells with anti-Calreticulin (top row) and anti-GM130 (middle row) antibodies. Control (no treatment) left panel versus BFA treatment (right panel, 50 ng/ml, 16 h) (scale bar: 10 μm). C: CerS6 localization was visualized by immunofluorescence confocal laser scanning microscopy (scale bar: 10 μm) by costaining cells with anti-CerS6 antibody, anti-GM130 antibody, and DAPI. D: Phosphorylation of CerS6 was assessed using lambda phosphatase treatment as described in Materials and Methods. Arrow: BFA-modified CerS6 band. Representative blots from three experiments are presented. E: Glycosylation of CerS6 was assessed using Endo-H or PNGase-F as described in Materials and Methods. Representative blots from three experiments are presented. DAPI, 4',6-diamidino-2-phenylindole; BFA, brefeldin A.

Our results described above suggest that CerS5 and/or CerS6, and their product C_16:0_-ceramide, may have a role in ER stress response. According to Protein Atlas database (Proteinatlas.org), the expression level of CerS6 is nearly 3 times that of CerS5 in HCT116 cells (29.6 nTPM and 10.5 nTPM, respectively). Thus, we elected to investigate the role of CerS6 in BFA-induced ER stress and UPR pathways. We analyzed the CerS6 protein using immunoblotting and found noteworthy changes in the abundance and the migration pattern of CerS6. While all the ER stress inducers that we used caused a slight decrease in CerS6 protein abundance, we observed a band shift in tunicamycin and BFA treated samples (Fig. 3A). CerS6 is known to be glycosylated (36, 37). Thus tunicamycin, a known glycosylation inhibitor (38), resulted in a faster migrating band likely due to decreased glycosylation of the protein (Fig. 3A, lane 3 vs. 4). Interestingly, BFA treatment resulted in slower migration of the CerS6 protein band, suggesting possible protein modifications following treatment (Fig. 3A, lane 5 vs. 6). We hypothesized that this modification may cause a change in CerS6 activity, resulting in increased C_16:0_-ceramide accumulation after BFA treatment (Fig. 2A). Given these data, we focused on BFA induced effects on CerS6.

To ensure that the concentration of BFA used indeed induced Golgi fragmentation as described in the literature (39), we performed confocal microscopy to observe changes to the ER and Golgi stained for calreticulin and GM130, respectively, following BFA treatment. We found that BFA treatment resulted in fragmentation of the Golgi and its collapse into the ER (Fig. 3B). Meanwhile, we found that the localization of CerS6 did not appear to change following BFA treatment, suggesting that the posttranslational modification observed was likely not due to a change in protein subcellular location (Fig. 3C). Next, we characterized the possible posttranslational modifications of CerS6 that are induced by BFA treatment. CerS6 is known to be phosphorylated (37). We first investigated the possibility of increased phosphorylation of the protein, which would produce a slower migrating band after BFA treatment. Treatment of lysates from control or BFA treated cells with recombinant lambda phosphatase caused a minor shift down in the CerS6 band consistent with known basal phosphorylation (37) (Fig. 3D, lane 1 vs. 2). In BFA treated lysates, lambda-phosphatase treatment caused a slight shift down but did not abrogate the main change in protein migration induced by BFA (Fig. 3D, lane 3 vs. 4), suggesting that the posttranslational modification responsible for the band shift following BFA treatment is not phosphorylation.

CerS6 is also known to be glycosylated (36), thus, we investigated possible additional glycosylation of the protein following BFA treatment. We treated cell lysates with two recombinant enzymes: Endo-H, which cleaves high mannose groups added in the ER (40), or PNGase-F, which also removes N-linked glycans added in the Golgi bodies (41). Endo-H caused faster CerS6 protein migration in untreated cells (Fig. 3E, lanes 1–2), consistent with removal of basal glycosylation, but caused little change in protein migration in BFA treated cells (Fig. 3E, lanes 3–4), indicating that BFA does not cause glycosylation in the ER. PNGase-F treatment of untreated cell lysate also caused faster protein migration, removing basal glycosylation (Fig. 3E, lanes 5–6). In BFA-treated lysates, PNGase-F resulted in band migration equal to that of the untreated cells (Fig. 3E, lanes 6–8), indicating that BFA treatment causes Endo-H resistant additional glycosylation of CerS6 occurring in the Golgi bodies.

### BFA treatment does not affect CerS6 activity

**Fig. 4:**
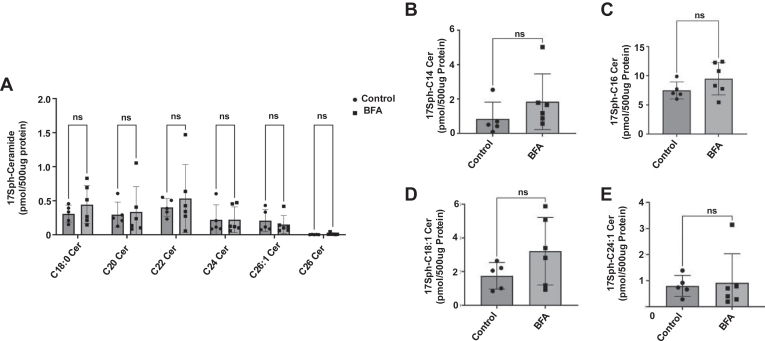
BFA does not increase CerS6 activity in situ. A–E: In situ CerS activity was measured by LC/MS using 17C-sphingosine labeling of HCT116 cells after treatment with BFA (50 ng/ml (178 nM), 16 h). Ceramide species with 17C-Sph backbone are shown. n = 3, three independent experiments with technical duplicates. Data represent mean ± SD. Statistical analysis was done by a two-way ANOVA with Tukey’s multiple comparison test. ns, nonsignificant *P* > 0.05. BFA, brefeldin A.

Our data show that BFA treatment induces C_16:0_-ceramide accumulation and additional CerS6 glycosylation, suggesting that this posttranslational modification may increase CerS6 activity. Therefore, we investigated this possibility using LC/MS in 17C-sphingosine labeling of HCT116 cells after treatment with BFA to measure the in situ activity of CerS6, or the flux of C17-Sph to C17-Sph-containing ceramides. To our surprise, we found that BFA treatment did not cause any significant change in 17C-Sph-Ceramides, suggesting that BFA does not increase CerS6 activity (Fig. 4A–E). We also analyzed generation of 17C-Sph-MonoHexosylCeramide and 17C-Sph-Sphingomyelin. Interestingly, we found that the levels of 17C-Sph containing C_16:0_-monohexosylceramide and C_16:0_-, C_20:0_-, and C_22:0_-sphingomyelins were significantly higher after BFA treatment (Supplemental Fig. S3A, B). These data suggest that BFA-induced additional glycosylation of CerS6 does not directly affect its activity in situ and 17C-Ceramides can be shuttled into monohexosylceramide and SM at a faster rate after BFA treatment. This is likely due to the collapse of Golgi, where the monohexosylceramide and SM generating enzymes are, onto the ER, bypassing the transport of ceramide to Golgi.

### Glycosylation at asparagine 18 is required for CerS6 activity

**Fig. 5:**
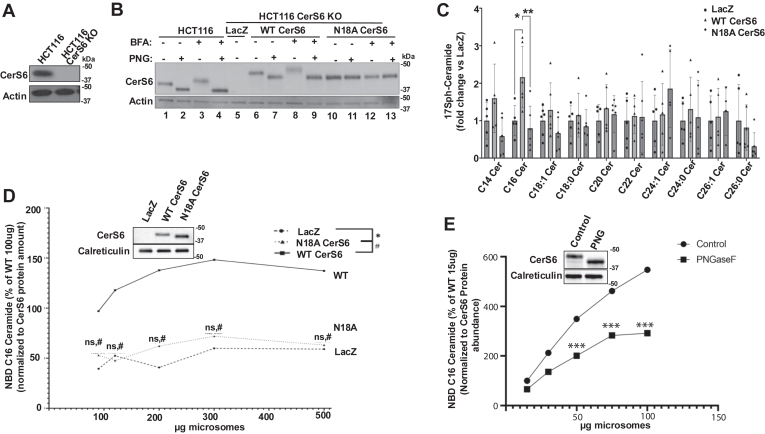
Basal glycosylation of CerS6 is required for CerS6 activity in vitro and in situ. A: CRISPR-Cas9 KO of CerS6 was performed in HCT116 cells as described in Materials and Methods. Validation of KO was assessed using Western blotting analysis of cell lysates. Representative blots from three experiments are presented. B: Stable expression of LacZ, WT CerS6, and N18A mutant CerS6 in CerS6 KO cells was assessed using Western blotting. Glycosylation of WT and N18A mutant was tested by treating cells with BFA (50 ng/ml (178 nM), 16 h) and treating cell lysates with PNGase-F as described in Materials and Methods. Representative blots from three experiments are presented. C: In situ CerS activity was measured by LC/MS using 17C-sphingosine labeling in HCT116 CRISPR-Cas9 KO cells stably expressing LacZ, WT CerS6, or N18A mutant CerS6. Ceramide species with 17C-Sph backbone are shown. n = 3, three independent experiments with technical duplicates. Data represent mean ± SD. Statistical analysis was done by a two-way ANOVA with Tukey’s multiple comparison test ∗*P* < 0.05, ∗∗*P* < 0.005. D: In vitro ceramide synthase activity of CerS5/6 was measured in HCT116 CRISPR-Cas9 CerS6 KO cells stably expressing either LacZ, WT CerS6, or N18A CerS6 using C_16:0_-CoA substrates. n = 3, three independent experiments with technical duplicates. Data represent mean ± SD. Statistical analysis was done by a two-way ANOVA with Tukey’s multiple comparison test. ∗*P* < 0.05 comparison between LacZ and N18A CerS6. #*P* < 0.05 comparison between WT CerS6 and N18A CerS6. ns, nonsignificant *P* > 0.05. D (inset) Expression levels of WT and N18A CerS6 was determined in the microsomes using Western blotting. E: In vitro ceramide synthase activity was measured in microsomes from HCT116 CRISPR-Cas9 CerS6 KO cells stably expressing WT CerS6 after either control or PNGase F treatment using C_16:0_-CoA substrates. n = 10, five independent experiments with technical duplicates. Data represent mean ± SD. Statistical analysis was done by a two-way ANOVA with Tukey’s multiple comparison test. ∗*P* < 0.05, ∗∗*P* < 0.01, ∗∗∗*P* < 0.005. ns, nonsignificant *P* > 0.05. E (inset). Removal of glycosylation of CerS6 determined by faster band migration in Western blotting analysis of microsomes subjected to PNGase F treatment. BFA, brefeldin A.

Our data indicate that BFA-induced additional CerS6 glycosylation does not affect its activity. However, we found that CerS6 is also basally glycosylated (Fig. 3E) in agreement with previous reports (36, 37). A possible role for CerS6 glycosylation was tested and glycosylation of CerS6 was concluded to be unnecessary for its activity. However, subsequent reports showed heterodimerization and homodimerization properties of CerS enzymes which can modulate their activity (42, 43). Therefore, we next tested whether basal glycosylation may play a role in CerS6 activity in better controlled experiments using more accurate HPLC-based CerS activity assay methods. To negate the possibility of homodimerization with the endogenous CerS6, we used reconstitution of CerS6 in a CerS6 KO cell line that is generated using CRISPR-Cas9 technology (Fig. 5A). CerS6 contains an asparagine residue at position 18 which is thought to be a potential glycosylation site (36). Therefore, we generated the CerS6 mutant with asparagine 18 mutated to an alanine (N18A) to prevent glycosylation at that residue. Next, we stably expressed control (LacZ), WT CerS6 (WT), or the mutant N18A CerS6 in CerS6 CRISPR/KO HCT116 cells to study the effect of glycosylation on CerS6 activity. We ensured that we could successfully modify and express WT and N18A CerS6 in HCT116 CerS6 KO cells. Our data show successful KO of CerS6 (Fig. 5B, lane 1 vs. 5) as well as successful reintroduction of WT CerS6 which responded to both BFA and PNGase-F treatment (Fig. 5B, lanes 6–9). Of note, the plasmid constructs used for reintroduction of WT or N18A mutant CerS6 included a linker and a C-terminal V5 tag which increased the protein’s molecular weight and led to a slower migrating band as compared to endogenous CerS6 (Fig. 5B, lanes 6–13). We also show expression of the N18A mutant CerS6 which migrated similar to the PNGase-F treated WT CerS6 expressing cells, indicating that this mutant can not to be glycosylated (Fig. 5B, lanes 10–13). Notably, the N18A mutant could not be glycosylated even at the basal level (Fig. 5B lane 10). To test whether the basal glycosylation plays a role in CerS6 activity, we measured the in situ CerS6 activity by performing LC/MS analysis in 17C-sphingosine labeled HCT116 CerS6 KO cells stably expressing LacZ, WT CerS6, or N18A CerS6. Our data show that WT CerS6 causes an increase in 17C-Sph-C_16_-ceramide compared to LacZ expressing cells (Fig. 5C). On the other hand, strikingly, our data show that the N18A CerS6 expressing cells produced significantly less 17C-Sph-C_16:0_-ceramide as compared to WT CerS6 expressing cells, suggesting lower CerS6 activity in the N18A CerS6 expressing cells (Fig. 5C). This suggests that basal glycosylation is required for CerS6 activity in the cells. Further, we measured the in vitro CerS6 activity in microsomes from the cells and found similarly that N18A CerS6 had significantly lower activity than WT CerS6 (Fig. 5D). Importantly, we show that there is little change in CerS6 in vitro activity with increasing amounts of microsomes in the LacZ and N18A CerS6 expressing cells, further suggesting the abrogation of CerS6 activity in these cell lines. This observed decrease in N18A CerS6 activity is present despite having more N18A CerS6 protein than the WT CerS6 microsomal fractions (Fig. 5D, inset). To validate that the inhibition of CerS6 activity in the N18A mutant is due to ablation of glycosylation specifically, we next subjected microsomes from the WT CerS6 expressing cells to PNGase F to remove glycosylation from the microsomal proteins, and performed in vitro activity assays. Consistent with our findings that the N18A mutant CerS6 expressing cells had decreased activity as compared to the WT CerS6 expressing cells, removal of glycosylation by PNGase F (Fig. 5E, inset) in the microsomes caused a significant reduction in the activity compared to the untreated microsomes (Fig. 5E). Taken together, these results suggest that CerS6 glycosylation at the position N18 is required for CerS6 activity.

### Glycosylation of CerS6 can influence down-stream cell signaling, but not ER-stress response

**Fig. 6:**
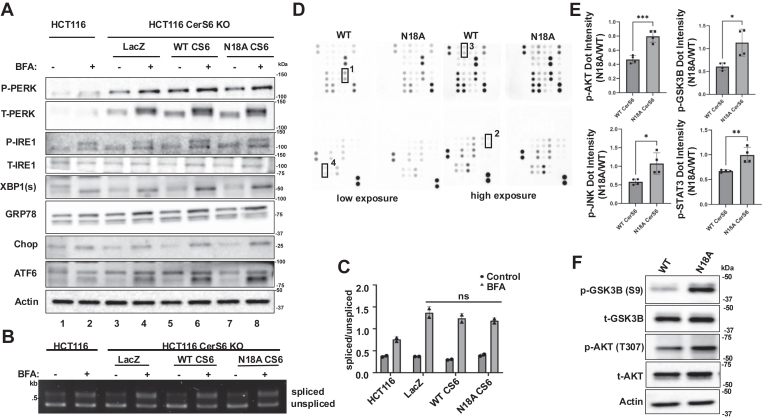
CerS6 glycosylation modulates kinase phosphorylation status. A: Protein abundance of UPR signaling proteins were measured by western blotting after treatment with BFA (50 ng/ml (178 nM), 16 h). Representative blots from three experiments are presented. B: XBP-1 mRNA splicing was analyzed using RT-PCR in the same samples as in A. C: Quantification of B using ImageJ analysis. Values were plotted as spliced divided by unspliced band intensity. n = 2, two independent experiments. Statistical analysis was done by a two-way ANOVA with Tukey’s multiple comparison test, ns nonsignificant *P* > 0.05. D: Protein phosphorylation or abundance was measured using the human proteome profiler in HCT116 CRISPR-Cas9 CerS6 KO cells expressing WT CerS6 or N18A mutant CerS6. 1. GSK3B (S9); 2. AKT1/2/3 (T308) 3. JNK1/2/3 (T183/Y185, T221/Y223); 4. STAT3 (Y705) E: Quantitation of D using ImageJ analysis. Values were plotted as N18A dot intensity divided by WT dot intensity. n = 4, two independent experiments. F: Changes in phosphorylation of GSK3B (S9) and AKT (T307) were measured by Western blotting in HCT116 CerS6 KO cells expressing WT or N18A mutant CerS6. Representative blots from three independent experiments are presented. BFA, brefeldin A; JNK, c-Jun N-terminal kinase; UPR, unfolded protein response; XBP1, X-box binding protein 1.

While we show that BFA treatment does not affect CerS6 activity via increased glycosylation, we observed changes in sphingolipids following BFA treatment (Fig. 2 and Supplemental Figs. S1 and S2). This, along with our data suggesting the importance of basal glycosylation in CerS6 activity, led us to investigate whether CerS6 basal glycosylation at N18 may play a role in ER stress response. We tested this by analyzing the changes in UPR signaling proteins following BFA treatment in our established cell lines (Fig. 5B). We found that in N18A mutant CerS6 expressing cells BFA-induced ER-stress response did not change as compared to the WT CerS6 expressing cells as indicated by similar increases in ER-stress related proteins following BFA treatment (Fig. 6A, lane pairs 5–6 and 7–8). Importantly, our data suggest that CerS6 is not involved in BFA-induced ER stress response given that there was no difference in ER-stress related proteins between BFA-treated LacZ expressing cells and the WT CerS6 expressing cells (Fig. 6A, lane pairs 3–4 and 5–6).

CerS6 may be important in the IRE1 arm of the ER-stress response (21); thus, we investigated this arm more closely and analyzed changes in spliced XBP-1 mRNA following BFA treatment in HCT116 and reconstituted cells. Consistent with the immunoblotting data (Fig. 6A), we found that N18A CerS6 expressing cells had similar levels of spliced XBP-1 mRNA as compared to the WT CerS6 expressing cells after BFA treatment (Fig. 6B, C). Importantly, we found that expression of LacZ, WT CerS6, and N18A CerS6 caused an increase in ER-stress markers and spliced XBP-1 mRNA as compared to the parental HCT116 cells, which may be due to forcible expression of the plasmids (Fig. 6A). Further, all transfected cells show an increase in ER stress markers upon BFA treatment, independent of CerS6 presence or activity, suggesting that CerS6 is not required for BFA-induced ER stress response. Therefore, we concluded that basal glycosylation of CerS6 is not required for ER-stress response.

Ceramide is known to modulate protein phosphatases and alter protein phosphorylation within the cell (44, 45, 46). We therefore used a Proteome Profiler to detect changes in the phosphorylation status of 37 human proteins involved in signaling events in cells expressing WT CerS6 and N18A CerS6. Of the 37 proteins tested, several proteins showed notable changes in their phosphorylation status or abundance in the WT CerS6 versus N18A CerS6 expressing cells including p-AKT 1/2/3 (S307), p-GSK3β (S9), p-JNK1/2/3 (T183/Y185, T221/Y223), and STAT3 (Y705) (Fig. 6D, E). Our results indicate that the N18A expressing cells have increased phosphorylation of AKT at threonine 307, GSK3β at serine 9, JNK at threonines 183 and 221 and at tyrosines 185 and 223, and STAT3 at tyrosine 705 (Fig. 6E). We confirmed the changes in phospho-GSK3β and phospho-AKT using immunoblotting analysis in WT and N18A CerS6 expressing cells and found, similarly, increase in phosphorylation (Fig. 6F). Given our previous data showing reduced CerS6 activity in the N18A mutant cells (Fig. 5C, D) as well as published work demonstrating a role for ceramide in GSK3β and β-catenin signaling (47, 48) as well as in AKT signaling (46, 49), it is possible that the reduction of ceramide in the N18A mutant cells may be responsible for the induction of GSK3β phosphorylation. Our data also indicate an increase in p-JNK1/2/3 (T183/Y185, T221/Y223) and STAT3 (Y705) in the N18A mutant cells (Fig. 6E). While each of these proteins are known to be involved in cancer signaling (50, 51, 52), there has been little work showing an association between ceramide or other sphingolipids with these specific pathways. Our work, therefore, may introduce a novel role for CerS6 generated C_16_-ceramide in JNK1/2/3 and STAT3 signaling pathways that should be further explored in future studies.

## Discussion

Our goal for this study was to investigate the possible role of sphingolipid metabolism in ER-stress response pathways. We found that induction of ER-stress by BFA causes accumulation of C_16:0_-ceramide and additional glycosylation of CerS6 which does not affect its activity. In addition, neither diminished CerS6 levels, nor its glycosylation influenced UPR signaling induction upon BFA treatment. However, very importantly, we show that, basal glycosylation of CerS6 is required for its activity. In contrast to previous studies which used overexpression of WT and mutant CerS6 (36), we used a robust CRISPR-Cas9 KO system followed by reintroduction of WT or N18A mutant CerS6 to study the effect of glycosylation on CerS6 activity. We found that loss of glycosylation at N18 reduces CerS6 activity both in vitro and in situ. Furthermore, we show that KO of CerS6 or abrogation of CerS6 glycosylation affects cellular signaling. These data not only provide vital insights into CerS regulation, but also shed light upon the importance of CerS posttranslational modifications in cellular signaling networks.

Mizutani *et al*. showed that CerS2, CerS5, and CerS6 are glycosylated on the N terminus, which faces the luminal space of the ER (36). They additionally found that the observed glycosylation of CerS6 was not essential for activity as shown by minimal changes in activity between cells overexpressing WT CerS6 and N18Q mutant CerS6 using a thin layer chromatography-based detection in the activity assay. Our data, on the other hand, suggest that basal glycosylation of CerS6 is required for its activity both in vitro and in situ. While Mizutani *et al*. overexpressed WT and mutant CerS6 in their experiments in the presence of functional endogenous CerS6, our work was performed using CerS6 CRISPR-Cas9 KO cells. Given that Laviad *et al*. show that CerS enzymes form heterodimers and homodimers which can affect their activity (42, 43), our robust KO system may offer further insight into the effect of glycosylation alone on CerS enzymes. For example, Laviad *et al*. show that functional CerS5 activity is modulated by catalytically inactive CerS5 enzyme; therefore, it is possible that the results presented by Mizutani *et al*. were complicated by the presence of endogenous CerS6 proteins. Our system, on the other hand, is not influenced by endogenous CerS enzymes, making it a robust system that can be used in future studies investigating modulation of CerS activity. We also show that CerS6 exhibits basal phosphorylation and glycosylation, as has been previously reported in the literature (36, 37). Sassa *et al*. showed that CerS2-6 are phosphorylated by CK2 in the C-terminal region and further reported that phosphorylation was critical for CerS2 activity whereas inhibition of phosphorylation in CerS3-6 only modestly affected enzymatic activity. We did not investigate the effect of CK2 induced phosphorylation on CerS6 activity in our CRISPR-Cas9 KO system here, but further investigations could be done in the future to assess this possibility.

While BFA induced accumulation of endogenous ceramide species, the flux of 17C-Sph to 17C-ceramide did not increase, suggesting that the increased endogenous ceramide levels were not a result of increased CerS activity. If analyzed alone, without the measurements of 17C-Sph containing 17C-HexCer and 17C-SM, one may consider the lack of changes seen in 17C-ceramide might be due to the substrate not reaching to the CerS6 active site on the ER, as 17C-Sph may be localized across a multitude of membranes within the cell rather than the ER alone. We found increases in the 17C-HexCer and 17C-SM levels following BFA treatment (Supplemental Fig. S3). Therefore, we believe that 17C-Sph reaches into the active site of CerS6 and is converted to 17C-ceramide before its conversion to 17C-HexCer and 17C-SM. The increase in 17C-SM and 17C-HexCer production following BFA treatment is likely due to the collapse of the Golgi into the ER, making ER-generated ceramide more available to sphingomyelin and glucosylceramide synthases, bypassing the transport of ceramide to Golgi steps. Additionally, the collapse of the Golgi into the ER may explain the additional CerS6 glycosylation following BFA treatment, as the ER-residing CerS6 enzyme becomes more available to Golgi-localized glycoysltransferases. This possibility could be further investigated as it may provide insights into the mechanisms underlying the relationship between ER stress response and sphingolipid metabolism.

Concomitant with accumulation of endogenous ceramides, BFA treatment caused decreases in SM species. Therefore, it is possible that the observed increase in ceramide species may be a result of increased sphingomyelin hydrolysis by activation of SMase enzyme(s). This possibility could be further tested in the future and perhaps could connect the source of ceramide elevation to the mediation of UPR via activation of a SMase.

Although we observed no change in CerS6 activity following BFA-induced additional glycosylation, we found that prevention of basal glycosylation in the mutant N18A CerS6 expressing cells decreased its activity both in vitro and in situ. 17C-Sph-C_16:0_-ceramide was significantly lower in N18A expressing cells as compared to WT expressing cells and was approximately equal to that of LacZ expressing cells. Notably, while our data from in situ CerS6 activity show N18A CerS6 activity as low as the LacZ expressing cells, our in vitro data also show that N18A CerS6 activity is not statistically different than that of the LacZ microsomes. Moreover, removal of glycosylation with PNGase F treatment causes a similar decrease in the WT CerS6 activity. Taken together, these data strongly suggest that glycosylation of CerS6 is required for its activity. Mizutani e*t al*., as well as AlphaFold (https://alphafold.edi.ac.uk) prediction place N18 in the luminal side of the ER (36, 53, 54). Further, Zelnik *et al*. recently proposed three distinct pockets in embedded within CerS2—cytoplasmic, midmembrane, and ER luminal—that work to accept the acyl-CoA and sphingoid substrates and bind them (55). It is possible, then, that glycosylation of Asparagine 18 in CerS6 may disrupt the binding of the acyl-CoA and sphingoid base within the ER-luminal pocket thereby decreasing its activity, as observed in our in situ and in vitro activity assays.

Our data suggest that CerS6 and its glycosylation does not affect ER stress induction and downstream signaling upon BFA treatment. However, several studies have shown a role for elevated ceramide and other sphingolipids in ER stress signaling. For example, a role for dihydroceramides in activating ATF6 via a transmembrane binding motif independent of unfolded proteins and dissociation of BiP was proposed (56). Mutation of this binding motif resulted in inhibition of ATF6 activation by dihydroceramide and dihydrosphingosine, but not through canonical ER stress induction by treatment with thapsigargin. Studies in multiple cancer types have also highlighted the link between sphingolipid metabolism and UPR. Exposure to C2-ceramide in adenoid cystic carcinoma cells induced UPR due to decrease Ca^2+^, and inhibition of CerS in the same cell lines inhibited UPR and rescued the cells from apoptosis (57). However, the opposite effect could also be seen in head and neck squamous cell carcinoma where knockdown of CerS6 and depletion of its product C_16:0_-ceramide induced UPR and subsequent apoptosis through decreased ER Ca^2+^, fusion of the ER and Golgi, and activation of ATF6 signaling (30, 58). Clearly, the role of sphingolipids in ER-stress signaling is complicated and warrants closer investigation.

Our results suggest that basal glycosylation of CerS6 in nonstressed cells is required for its activity as it causes changes in the endogenous CerS6 activity and can subsequently modulate other crucial cellular signaling pathways. We found that N18A mutant CerS6 expressing cells had an increase in GSK3β and AKT phosphorylation at serine 9 and threonine 307, respectively, as compared to the WT CerS6 expressing cells. This finding is consistent with previous reports from Coant *et al*. and Garcìa-Barros *et al*. that show a role of ceramide in the regulation of GSK3β and β-catenin via modulation of AKT (47, 48). It is possible that the decreased ceramide production in the N18A CerS6 expressing cells may be the cause of the observed increase in GSK3β phosphorylation. Given that there is a well-established role for ceramide in Akt signaling via PP2A (59, 60), it is likely that the observed modulation in GSK3β phosphorylation is via ceramide-mediated changes in PP2A and Akt dephosphorylation. The role of Akt and its downstream targets in cancer cell proliferation and progression is well-established (47, 61). Our work presents CerS6 and specifically CerS6 glycosylation, as a potential therapeutic target for the modulation of GSK3β and AKT signaling in cancer models.

In summary, our results show a previously underappreciated mode of CerS6 regulation through its glycosylation. Although this glycosylation was dispensable for ER-stress pathway induction, it is required for basal CerS6 activity and can regulate downstream signaling modules important for biological functions.

## Data availability

All the data are contained within the manuscript. Data reported in the present study can be shared upon request to Dr Can E. Senkal, Virginia Commonwealth University School of Medicine, Department of Biochemistry and Molecular Biology, can.senkal@vcuhealth.org.

## Supplemental data

This article contains supplemental data.

## Conflict of interest

The authors declare that they have no conflicts of interest with the contents of this article.
