# Supplementary material for: Glycosylation of ceramide synthase 6 is required for its activity
Source: J Lipid Res. 2024 Nov 26;66(1):100715. doi: 10.1016/j.jlr.2024.100715 (PMC11732463; doi:10.1016/j.jlr.2024.100715)
Supplement: Supplemental information [file mmc1.pdf]

SUPPLEMANTAL INFORMATION

Glycosylation of Ceramide Synthase 6 is Required for Its Activity

Alexandra J. Straus[1,2], Grace Mavodza[1], Can E. Senkal[1,3,*]

[1]Department of Biochemistry and Molecular Biology; [2] C. Kenneth and Dianne Wright Center for Clinical and Translational Research; [3] Massey Comprehensive Cancer Center, Virginia Commonwealth University School of Medicine, Richmond, Virginia, USA

Corresponding Author: Can E. Senkal, Email: can.senkal@vcuhealth.org, phone number: 804-828-2620, address: 1101 Marshall Street, Sanger Hall, Room 2040D, Richmond VA 23298

Supplemental Figure 1

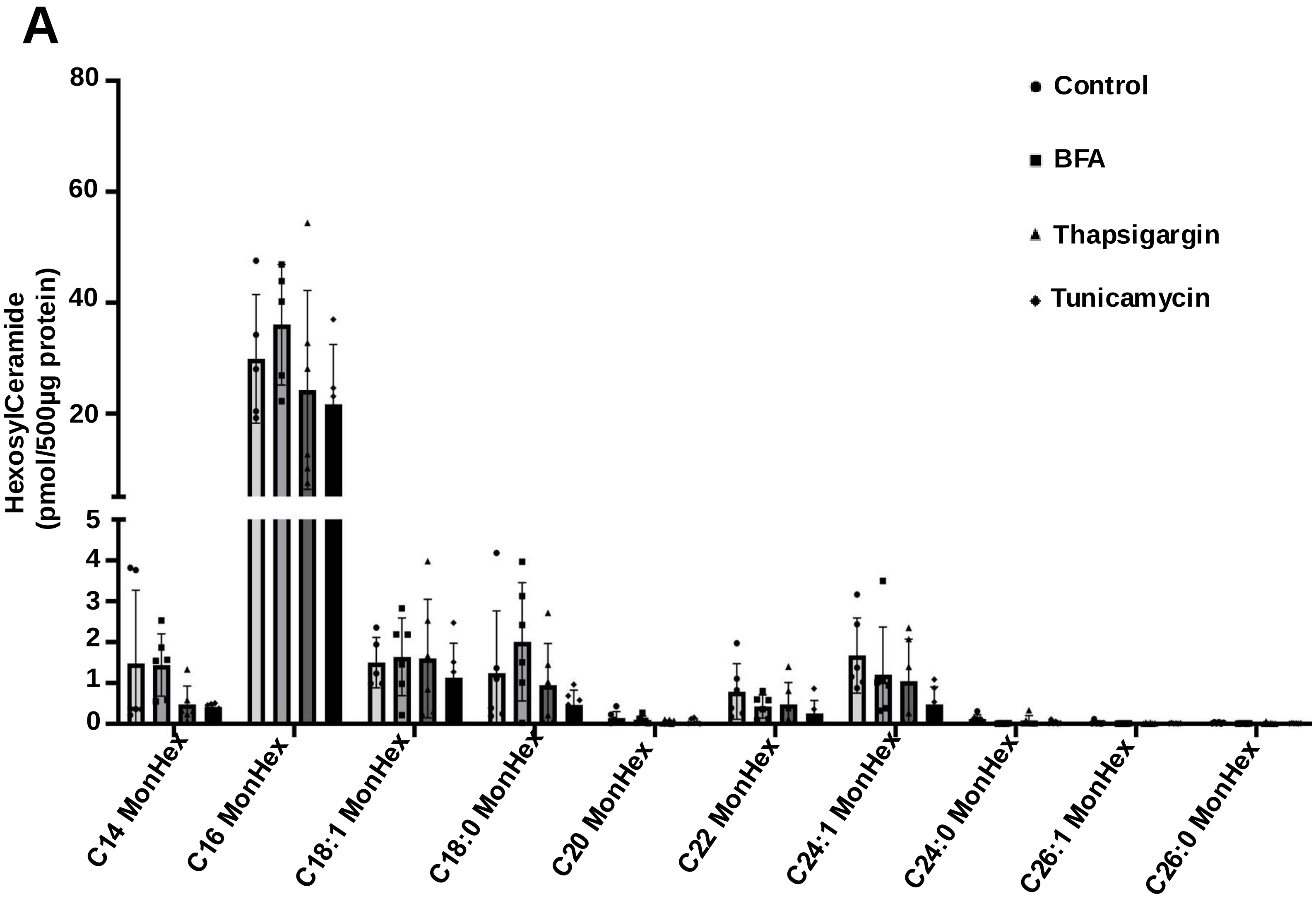

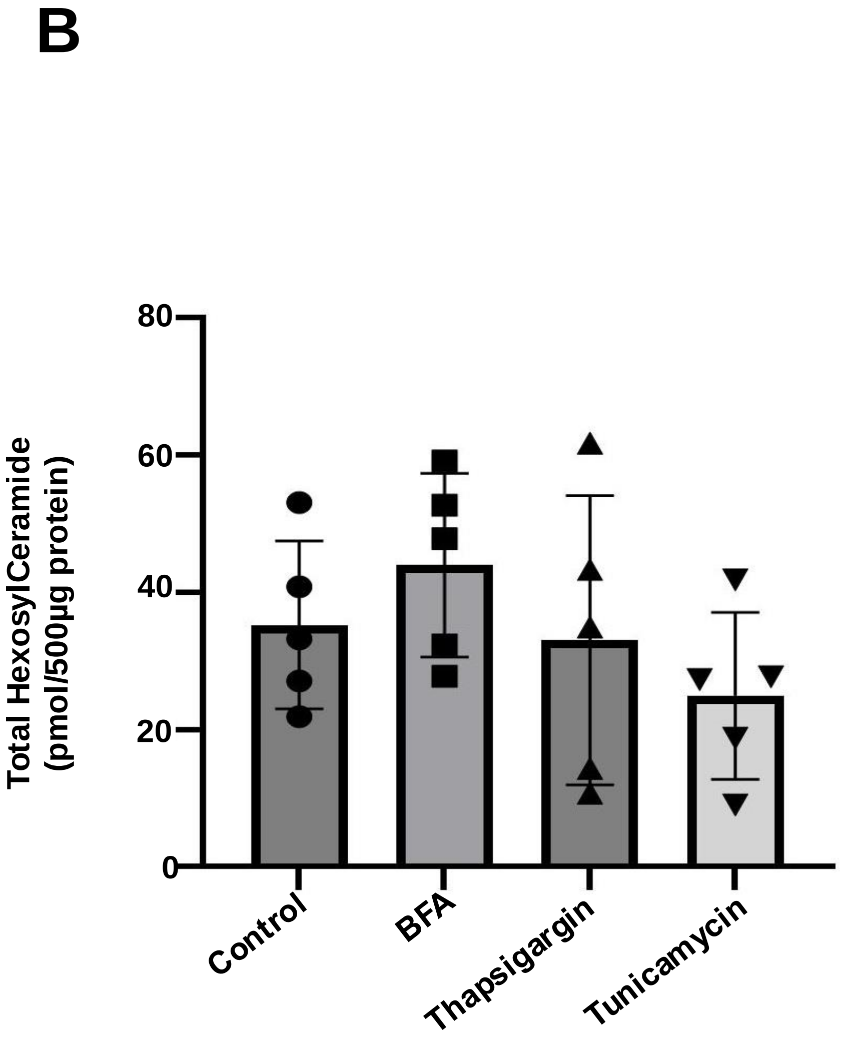

Supplemental Figure 1. BFA Treatment Does Not Affect MonoHexosylCeramide Levels. A-B. Sphingolipid levels were measured in HCT116 cells by LC/MS as described in Materials and Methods. Cells were treated with BFA (50ng/mL (178 nM)), Thapsigargin (50nM), or Thapsigargin (0.5μg/mL (768 nM)) for 16 hrs. Lipid abundance was normalized to 500μg protein as measured by a Bradford assay. n=3 (Three independent experiments with technical duplicates) Data represent mean ± SD. Statistical analysis was done by a two-way ANOVA with Tukey's multiple comparison test *p<.05, **p<.005, ***p<.001.

Supplemental Figure 2

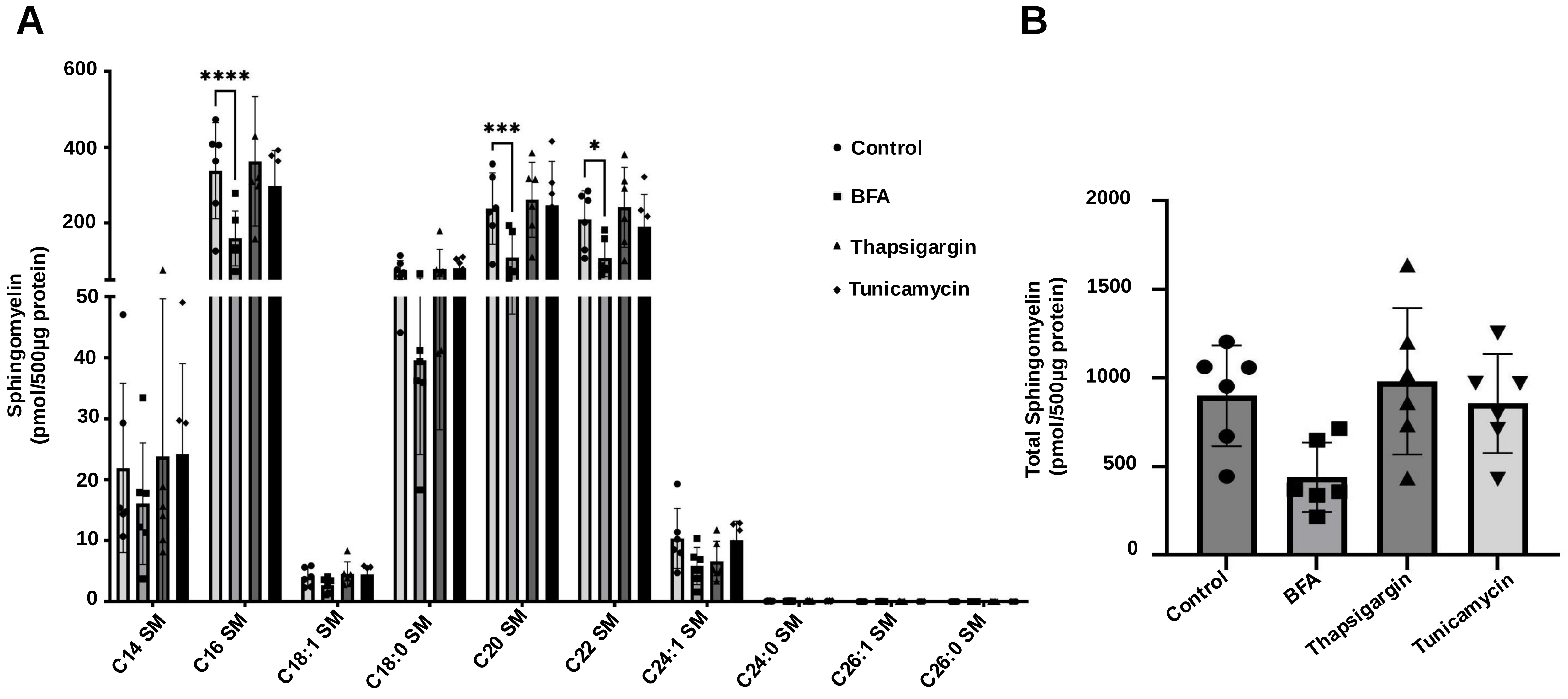

Supplemental Figure 2. BFA Treatment Reduces $C_{16:0}$-, C_{20}-, and C_{22}-Sphingomyelin Levels. A-B. Sphingolipid levels were measured in HCT116 cells by LC/MS as described in Materials and Methods. Cells were treated with BFA (50ng/mL (178 nM)), Thapsigargin (50nM), or Thapsigargin (0.5µg/mL (768 nM)) for 16 hrs. Lipid abundance was normalized to 500µg protein as measured by a Bradford assay. n=3 (Three independent experiments with technical duplicates) Data represent mean ± SD. Statistical analysis was done by a two-way ANOVA with Tukey's multiple comparison test *p<.05, **p<.005, ***p<.001.

Supplemental Figure 3

A

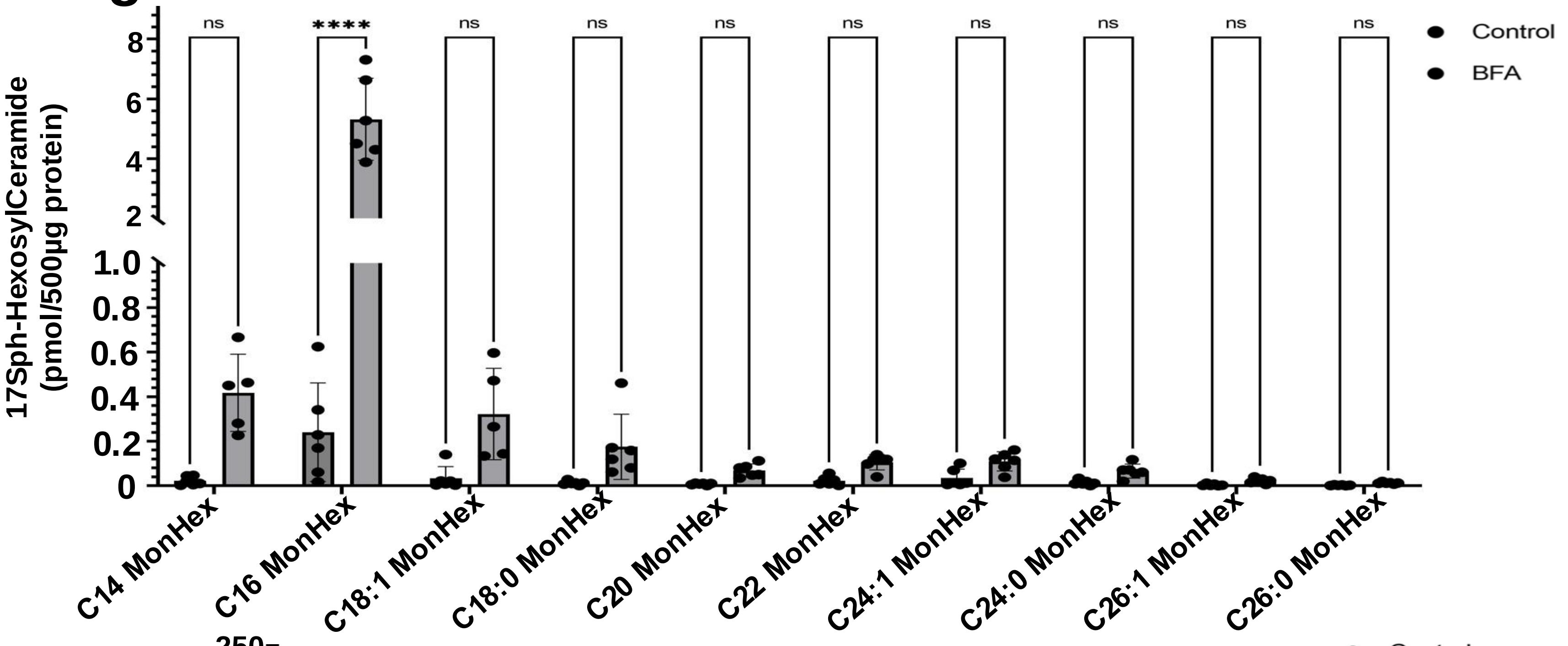

B

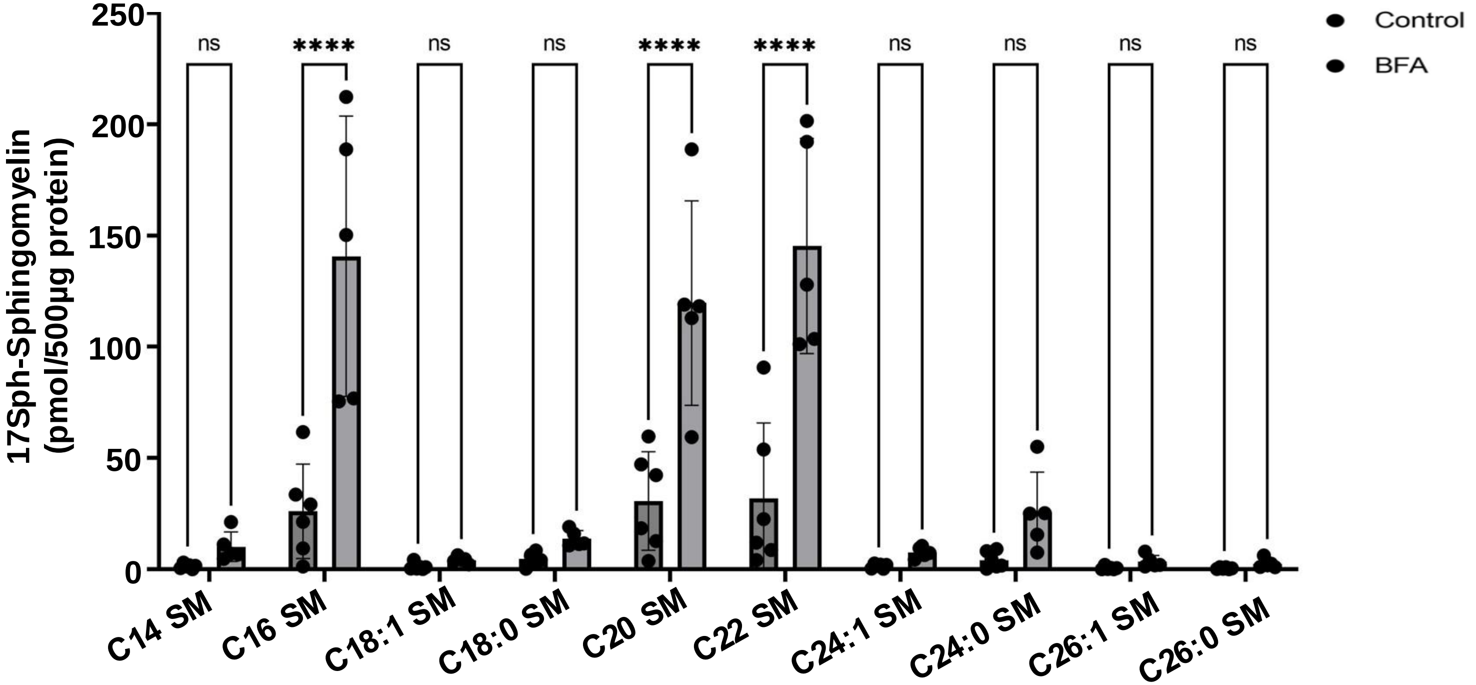

Supplemental Figure 3. BFA Treatment Causes Accumulation of 17C-Sph-$C_{16:0}$-MonoHexosylCeramide and 17C-Sph-C_{16}-, C_{20}-, and C_{22}-Sphingomyelins A-B. Incorporation of 17C-Sph into monohexosylceramides and SM were measured by LC/MS using 17C-sphingosine labeling of HCT116 cells after treatment with BFA (50ng/mL (178 nM), 16hrs). MonoHexosylCeramide and SM species with 17C-Sph backbone are shown. n=3, three independent experiments with technical duplicates. Data represent mean ± SD. Statistical analysis was done by a two-way ANOVA with Tukey's multiple comparison test. *p<.05, **p<.005, ***p<.001.
